# Overcoming the rise in local deposit resistance during electrophoretic deposition *via* suspension replenishing

**DOI:** 10.3389/fchem.2022.970407

**Published:** 2022-08-26

**Authors:** Prabal Tiwari, Noah D. Ferson, David P. Arnold, Jennifer S. Andrew

**Affiliations:** ^1^ Department of Materials Science and Engineering, University of Florida, Gainesville, FL, United States; ^2^ Department of Electrical and Computer Engineering, University of Florida, Gainesville, FL, United States

**Keywords:** electrophoretic deposition kinetics, colloidal processing, scalable nanomanufacturing, materials assembly, alumina

## Abstract

Nanomaterials have unique properties, functionalities, and excellent performance, and as a result have gained significant interest across disciplines and industries. However, currently, there is a lack of techniques that can assemble as-synthesized nanomaterials in a scalable manner. Electrophoretic deposition (EPD) is a promising method for the scalable assembly of colloidally stable nanomaterials into thick films and arrays. In EPD, an electric field is used to assemble charged colloidal particles onto an oppositely charged substrate. However, in constant voltage EPD the deposition rate decreases with increasing deposition time, which has been attributed in part to the fact that the electric field in the suspension decreases with time. This decreasing electric field has been attributed to two probable causes, (i) increased resistance of the particle film and/or (ii) the growth of an ion-depletion region at the substrate. Here, to increase EPD yield and scalability we sought to distinguish between these two effects and found that the growth of the ion-depletion region plays the most significant role in the increase of the deposit resistance. Here, we also demonstrate a method to maintain constant deposit resistance in EPD by periodic replenishing of suspension, thereby improving EPD’s scalability.

## Graphical Abstract

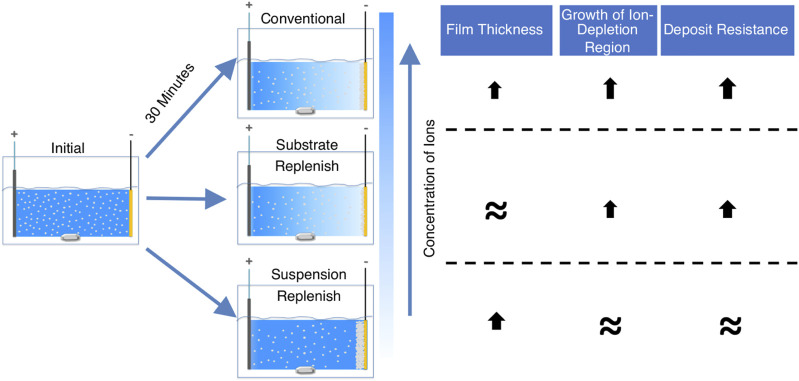

## Highlights

1) Suspension replenishment enables constant deposit resistance with time.
2) Ion-depletion causes a decrease in the electric field during electrophoretic deposition.
3) Film formation plays an insignificant role in increasing deposit resistance.
4) Electric field can be maintained constant *via* ion-replenishing.

## 1 Introduction

The growth of nanotechnology is contingent upon further advancements in nanomanufacturing technologies (Philp and Fraser Stoddart, 1996; Whitesides and Grzybowski, 2002; Gates et al., 2005; Biswas et al., 2012; Bauer et al., 2018). Nanomanufacturing techniques can be broadly classified into top-down and bottom-up approaches or some combination of the two (Philp and Fraser Stoddart, 1996; Whitesides and Grzybowski, 2002; Gates et al., 2005; Merkel et al., 2010; Biswas et al., 2012; Pascall et al., 2014; Vogel et al., 2015; Jesse et al., 2016; Liu et al., 2016; Isaacoff and Brown, 2017; Fu et al., 2018; Yin et al., 2020). Top-down approaches involve cutting down macro-sized materials into nanomaterials with the help of external stimuli. Some examples of top-down approaches are lithography, molding, and milling. On the other hand, bottom-up approaches involve the assembly of atoms/molecules/colloidal particles into a larger nanostructure driven by interaction forces between them and/or external stimuli. Some examples of bottom-up approaches are atomic layer deposition, sol-gel nanofabrication, self-assembly, vapor deposition, Langmuir-Blodgett assembly, electric-field-assisted assembly, and capillary-force-assisted assembly. Both top-down and bottom-up approaches have benefits and limitations of their own. Top-down approaches have the advantage of precision and repeatability (Biswas et al., 2012; Jesse et al., 2016). However, they suffer from high capital and operating costs, low scalability, and diffraction limits of the electromagnetic wave being used which limits the feature size in lithography (Gates et al., 2005; Merkel et al., 2010; Biswas et al., 2012; Fu et al., 2018). Bottom-up approaches, on the other hand, have the advantage of high scalability, relatively low cost, the ability to fabricate structures upon non-planar substrates, and compatibility with organic materials (Philp and Fraser Stoddart, 1996; Whitesides and Grzybowski, 2002; Biswas et al., 2012; Vogel et al., 2015; Isaacoff and Brown, 2017; Yin et al., 2020). However, bottom-up approaches suffer from limited repeatability and a lack of control over the process. Thus, improvements and innovations in both top-down and bottom-up approaches are required to develop the next generation of nanomanufacturing technologies.

One promising bottom-up technique that is gaining attention in research labs and industry because of its scalability, cost-effectiveness, tunability, simple setup, and versatility in depositing a wide range of materials over many substrate shapes is electrophoretic deposition (EPD) (Besra and Liu, 2007; Dickerson and Boccaccini, 2011; Kalinina and Pikalova, 2019; Sikkema et al., 2020; Atiq Ur Rehman et al., 2021). EPD assembles charged colloidal particles onto an oppositely charged substrate under the application of an externally applied electric field. Due to its advantages, EPD has been used to form 2-D and 3-D assemblies of various classes of materials such as metals, ceramics, organics, and biomaterials onto planar, non-planar, and patterned substrates (Sarkar and Nicholson, 1996; Van der Biest and Vandeperre, 1999; Biest and Vandeperre, 2002; Zhitomirsky, 2002; Sarkar et al., 2004; Besra and Liu, 2007; Corni et al., 2008; Novak and König, 2009; Boccaccini et al., 2010b, 2010a; Boccaccini and Dickerson, 2013; Chavez-Valdez et al., 2013; Guo et al., 2015; Diba et al., 2016; Ma et al., 2018; Kalinina and Pikalova, 2019; Obregón et al., 2019; Hu et al., 2020; Lim et al., 2021). However, the underlying mechanisms and kinetics of EPD are still not well-understood (Dickerson and Boccaccini, 2011; Fuseini and Zaghloul, 2022).

Although the first-documented observation of electrophoresis was done by the French chemist Gautherot in 1801, it took more than a century before a theoretical framework for the kinetics of EPD were established by Hamaker in 1940 (Hamaker, 1940; Besra and Liu, 2007). According to Hamaker’s equation (Equation 1), EPD yield (m) depends on a sticking parameter (*f*) which is the ratio of the number of particles that adhere to the substrate and become part of the deposit to the total number of particles reaching the substrate, particle concentration (C_s_), electrophoretic mobility of the particle being deposited (µ), area of the substrate (A), effective electric field in the suspension (E), and time (t):

$$m=fC_{s}\mathrm{\mu AEt}$$ (1)

Later it was observed that some of these factors do not necessarily remain constant but can decrease as a function of time, which can lead to a plateau in EPD yield (Sarkar and Nicholson, 1996; Anné et al., 2004; van der Biest et al., 2004; Wang et al., 2004; Stappers et al., 2008; Ferrari and Moreno, 2010). The particle concentration in the suspension decreases as particles deposit on the substrate, or as they settle out of the suspension. The electrophoretic mobility of the particles has also been shown to decrease during EPD due to a shift in the pH of the suspension (Kershner et al., 2004; Tiwari et al., 2020). Finally, the effective electric field in the suspension has been observed to decrease in the suspension during constant-voltage EPD (Zhang et al., 1994; Sarkar and Nicholson, 1996). The factors responsible for causing a decrease in particle concentration and particle electrophoretic mobility are well-understood and can be controlled (Sarkar and Nicholson, 1996; Besra and Liu, 2007; Tiwari et al., 2020). However, there is currently no consensus on the factor(s) that cause the decrease in electric field in the suspension (De and Nicholson, 2004; Kershner et al., 2004; Besra et al., 2010; Mishra et al., 2010; MISHRA et al., 2013; Tiwari et al., 2020).

**FIGURE 1:**
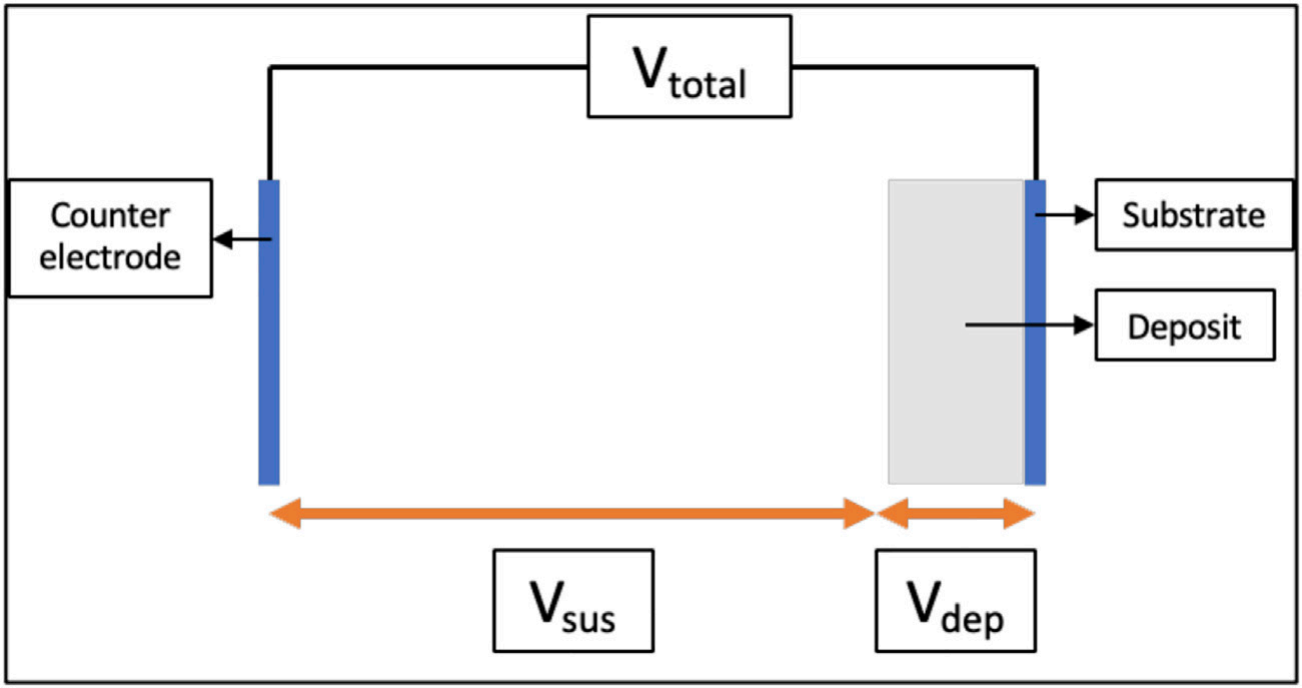
Schematic showing voltages across a typical electrophoretic deposition cell used in constant-voltage mode.

In constant-voltage EPD, the total applied voltage difference (V_total_) between the substrate and the counter-electrode is maintained constant using a power supply as shown with the help of a schematic in Figure 1. The V_total_ in the EPD cell is distributed as the sum of the voltage drop across the suspension (V_sus_) and the voltage drop across the deposit (V_dep_). The corresponding electric fields in the suspension (E_sus_) and the deposit (E_dep_) are not necessarily the same, or constant with time. In current literature, an increase in V_dep_ and corresponding E_dep_ with time has been observed (Sarkar and Nicholson, 1996; Negishi et al., 2005; van Tassel and Randall, 2006b; Stappers et al., 2008). This increase in V_dep_ was widely attributed to the rise in local deposit resistance (R_dep_) during EPD, which will be discussed in-depth in the next section. Consequently, there is a decrease in V_sus_, and in the corresponding E_sus_ (Negishi et al., 2004, 2005). Therefore, to understand and be able to better control the electric field on the deposit as well in the suspension, it is vital to understand the root cause behind the rise in local deposit resistance as a function of time.

There have been different explanations put forward to justify the increase in local deposit resistance during EPD (Boccaccini and Dickerson, 2013). Some researchers suggested that the increase in deposit resistance is caused because the thickness of the deposit is increasing with time (Sarkar and Nicholson, 1996). The hypothesis for this justification is that the resistivity of the deposit is higher than that of the suspension, and hence, an increase in deposit thickness leads to an increase in deposit resistance (Sarkar and Nicholson, 1996). However, further studies contradicted this claim by showing that the resistance of the deposit remains the same before and after the deposition, suggesting there must be another factor causing the rise in deposit resistance during EPD (Argirusis et al., 2006; Stappers et al., 2008; Schneider et al., 2010).

Further theoretical and experimental studies pointed to the electrochemical consumption of ions on the deposit electrode as the reason for the rise in deposit resistance during EPD (van Tassel and Randall, 2006b; Stappers et al., 2008; Hu et al., 2019). In a series of papers, Tassel et al. put forward a theory to explain the formation and stabilization of an ‘ion-depleted conduction region’ on the deposit electrode during constant-current EPD (De and Nicholson, 2004; van Tassel and Randall, 2006a, 2006b, 2007a, 2007b, 2007c). They showed that during EPD, a high resistivity ‘ion-depleted conduction region’ forms and grows due to the consumption of ions on the deposit electrode. In a typical electrochemical cell, this region will not be stable because of the immediate onset of convection currents that supply ions to this region. However, they argued that in an EPD cell, this region is stabilized against convection due to the buffering action of particles i.e., adsorption/desorption equilibria of ions from the particle surface present in the ion-depleted region. Thus, this region is confined to the deposited particle layer. The conduction of ionic current through this high resistivity ‘ion-depleted conduction region’ can proceed even above the equilibrium limit current and leads to a very high R_dep_ and E_dep_. On the other hand, in constant-voltage EPD, the electrochemical consumption of ions on the deposit electrode will also lead to the formation and growth of a stable ion-depleted conduction region. The only difference is that the current cannot exceed the equilibrium limit current in constant-voltage EPD. To study the role of the ion-depleted region on deposit resistance during constant-voltage EPD, Stappers et al. measured the deposit impedance as a function of time in a constant-voltage EPD *via* impedance spectroscopy (Stappers et al., 2008). They reported that the deposit impedance increases as deposition proceeds but returned to the original value after the deposition was stopped. Moreover, when they paused the deposition and replaced the particle suspension with the supernatant solution (without particles) and then resumed the deposition, the deposit impedance continued to increase. These two observations suggest that the deposit impedance is a function of the electrochemical depletion of ions on the deposit electrode rather than the growth of high-resistivity particle film. However, they were unable to maintain a constant deposit impedance and hence constant electric field, likely because they are merely recirculating the suspension from a reservoir where the ionic concentration was being depleted with time.

**FIGURE 2:**
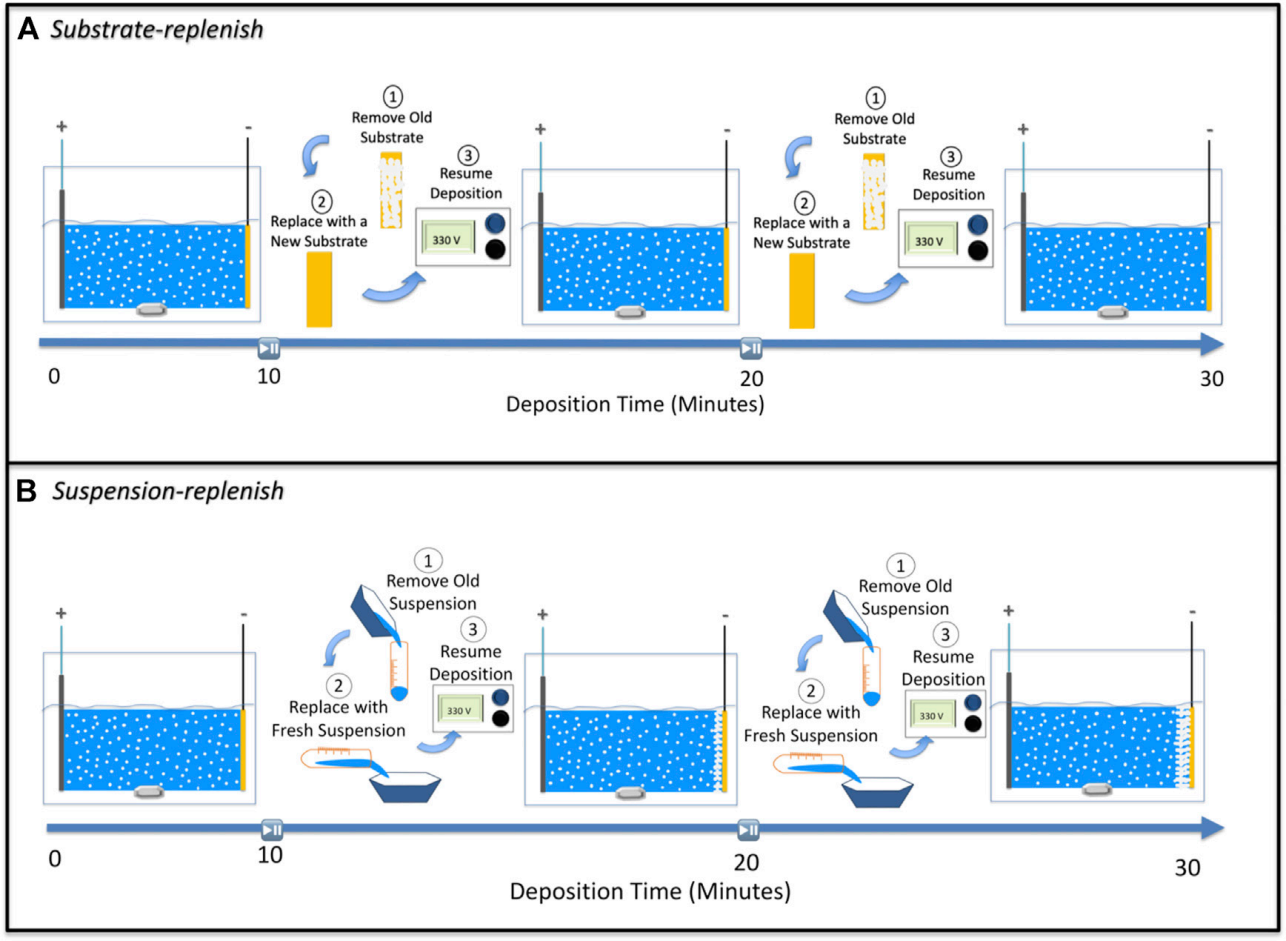
Schematic showing steps involved in **(A)** substrate-replenish electrophoretic deposition and **(B)** suspension-replenish electrophoretic deposition.

We hypothesized that instead of recirculating the suspension as per the study by Stappers et al., replenishing the suspension using a suspension-replenish EPD approach (Figure 2B) it would be possible to hinder the growth of the ion-depletion region and maintain constant deposit resistance as it will keep the ionic concentration constant during EPD. To confirm our hypothesis, in this study, we isolated and compared the individual effects of the growing deposit and the growing ion-depletion region on the increase in deposit resistance during constant-voltage EPD of alumina particles. We accomplished this by developing two modified EPD approaches (details provided in the experimental section), where either the substrate (along with the deposited film) or the suspension was replenished (Figure 2). Substrate-replenish EPD (Figure 2A) allowed us to periodically restart the growth of the deposit onto a fresh substrate while allowing the ion-depletion region to grow unhindered. On the other hand, suspension-replenish EPD (Figure 2B) allowed us to periodically restart the growth of the ion-depletion region by replenishing the ions and particles in the suspension while letting the deposit grow unhindered. We found that the growth of the ion-depletion region and not the deposit growth causes the increase in deposit resistance. We also found that the deposit resistance remained nearly constant during the suspension-replenish EPD. Thus, confirming our hypothesis that the growth of the ion-depletion region can be hindered, and deposit resistance can be kept constant *via* replenishment of ions using suspension-replenish EPD, which then helps maintain a near-constant electric field in the suspension.

For future work, improvements to suspension-replenish EPD can be achieved using a continuous-flow setup with automatic adjustments based on a live feedback mechanism to maintain constant particle concentration, pH, and conductivity in the EPD cell. Moreover, when measuring the electric field during EPD, one must be aware that the presence of the mulitmeter probe has the potential to impact the experiment. To overcome these challenges, computational tools can be utilized. Simulations of EPD experiments at varying scales will further help us visualize and understand the local and bulk electrochemical changes occurring during EPD.

## 2 Experimental details

### 2.1 Suspension preparation and characterization

α-alumina nanoparticles (> 99% purity, 80 nm diameter, 3.97 g/cm^3^ density) were purchased from US Research Nanomaterials, Inc., USA. A positively charged suspension of 0.25 volume % α-alumina nanoparticles was prepared in ethanol (200 proof ethanol, Acros Organics) by using hydrochloric acid (Technical HCl, Fischer Chemical) as the dispersing agent. The suspension was sonicated using an ultrasonicator (Branson SFX 550 probe ultra-sonicator). Effective pH (pHe) measurements were made using the ASTM D6423-19 standard protocol using a Thermo Scientific 5107 BNMD No-Cal pH/Automated Temperature Compensation combination electrode connected to a Thermo Scientific Orion Star A111 bench-top pH meter (Fuel and Engines, 1999). pHe measurements were recorded after 30 s of immersion in a stirred suspension. A Malvern Panalytical Zetasizer was used to measure the electrophoretic mobility and zeta potential of the nanoparticles in suspension. The Hückel model was used to calculate the zeta potential from particle mobility (Hunter, 2001). The suspension, with a pHe of 1.70 +/- 0.04 obtained by mixing 0.4 ml of 0.02 M HCL in ethanol solution with 19.6 ml of ethanol having particle mobility and zeta potential of 0.74 +/- 0.07 µm cm/V.s and 53.90 +/- 4.79 mV respectively, was used as the starting suspension for all depositions.

### 2.2 Electrophoretic depositions

**FIGURE 3:**
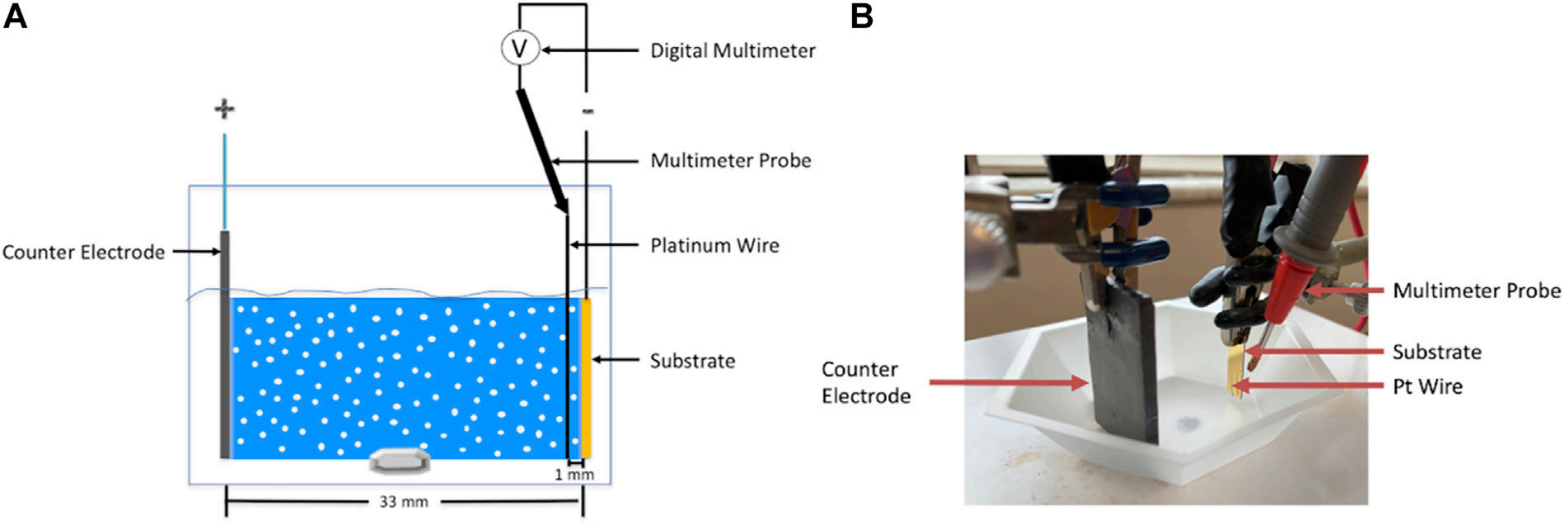
**(A)** Schematic and **(B)** the corresponding photograph of the electrophoretic deposition setup.

Figure 3 shows a schematic and a corresponding photograph of the electrophoretic deposition setup that was used to perform the deposition of alumina nanoparticles using an applied electric field of 100 V/cm. Here, a hexagonal weighing boat was used to contain 20 ml of suspension. A magnetic stir rod and plate were used to stir the suspension during deposition. The electric field was applied by connecting a counter electrode (graphite) and a substrate placed 33 mm apart in the suspension to the positive and negative terminals of the power supply, respectively. To fabricate the substrate, a 10 nm titanium adhesion layer was sputtered onto a 100 4-inch prime-grade silicon wafer followed by sputtering of 100 nm gold layer using a KJL CMS-18 sputtering instrument. The wafer was then diced into 6 mm (width) × 20 mm (height) substrates using an ADT 7100 dicing saw. To image the EPD films, a cross-section of the film was prepared using an FEI Helios Nanolab 600 dual-beam focused ion beam (FIB)/Scanning Electron Microscope (SEM) using methods described elsewhere (Langford and Petford-Long, 2001). Imaging of the cross-section was performed using the same instrument; the cross-section was tilted 45° relative to the electron beam and dynamic focus was used to keep the entire cross-section in focus.

#### 2.2.1 Substrate replenish electrophoretic deposition

In this substrate replenish EPD approach, we ran 30-min depositions starting with a fresh suspension of alumina nanoparticles at 100 V/cm while pausing the deposition at 10-min and 20-min time points to remove the substrate covered with deposit and to introduce a fresh substrate (without any deposit). The old suspension was collected in a centrifuge tube whilst the substrate is being replenished. After the substrate was replaced, the old suspension was reintroduced into the EPD boat followed by resumption of deposition (Figure 2A).

#### 2.2.2 Suspension replenish electrophoretic deposition

In this suspension replenish EPD approach, we performed 30-min depositions starting with a fresh suspension of alumina nanoparticles at 100 V/cm while pausing the deposition at 10-min and 20-min time points to remove the old suspension and introduce a fresh suspension followed by resumption of the deposition (Figure 2B).

### 2.3 Deposit resistance measurement

The deposit resistance was calculated *via* Ohm’s law using the voltage difference measured between the substrate (deposit electrode) and a Pt wire (0.1 mm diameter) submerged at a distance of 1 mm parallel to the center of the substrate and the current value that was recorded from the power supply. A Siglent SDM 3055 digital multimeter and multimeter probe were used to measure the voltage difference as shown with the help of a schematic in Figure 3. The Pt wire was electrically connected to the multimeter probe with copper (Cu) tape. A distance of 1 mm was chosen as it allowed us to keep the Pt wire as close to the substrate as possible without disrupting the film growth since our films did not grow thicker than 1 mm. The voltage readings were automatically recorded using the Easy DMM software (Siglent).

## 3 Results and discussion

### 3.1 Deposit resistance during conventional electrophoretic deposition

**FIGURE 4:**
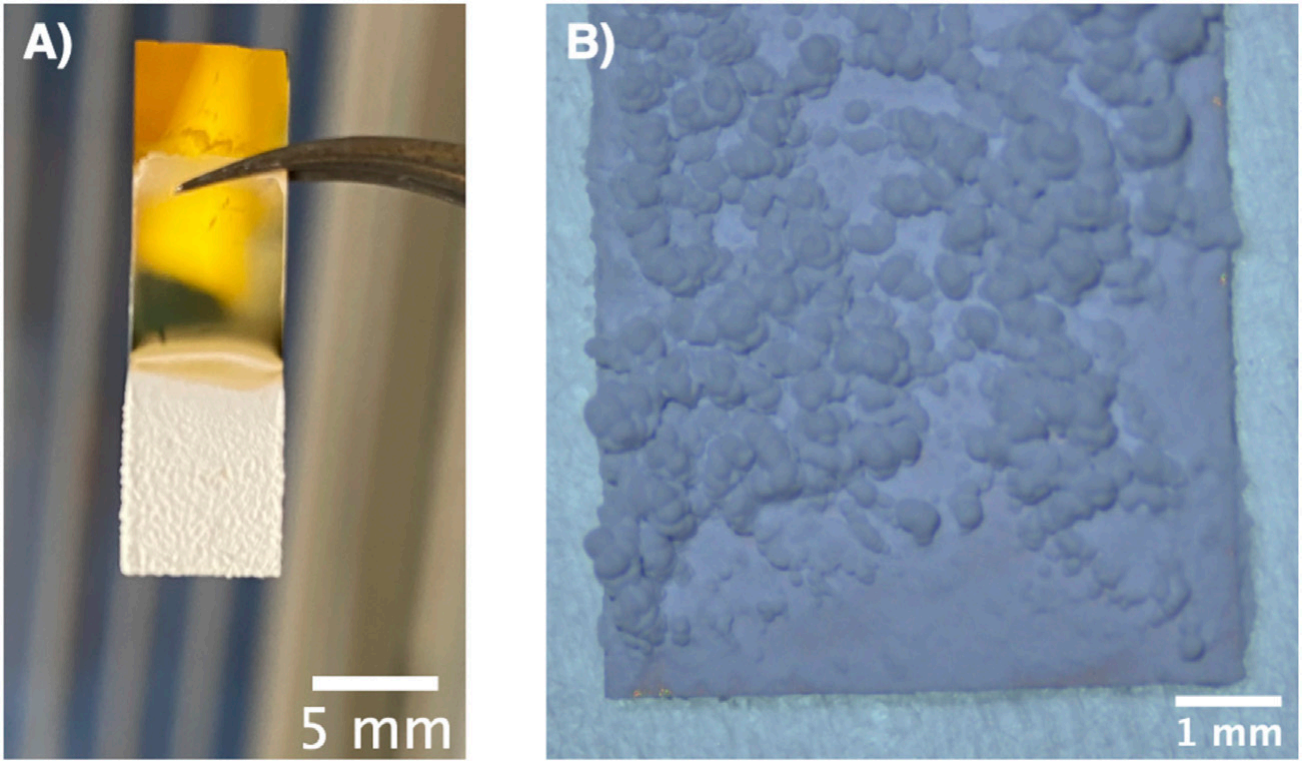
**(A)** Photograph and **(B)** optical micrograph of a typical ⍺-alumina nanoparticles film deposited *via* electrophoretic deposition.

**FIGURE 5:**
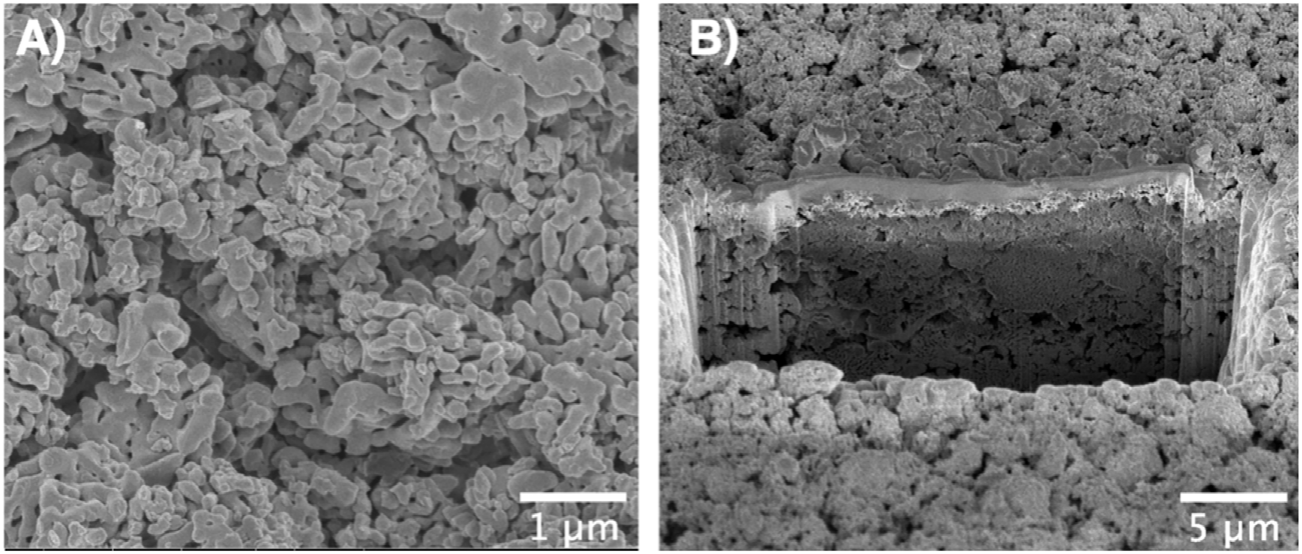
**(A)** Top-view SEM image and **(B)** Cross-sectional SEM image of the film prepared *via* FIB showing the porous structure of the EPD film.

Figure 4 shows a photograph and an optical micrograph of a typical film obtained *via* electrophoretic deposition of α-alumina nanoparticles. Here, full coverage of the submerged part of the substrate with the particles is seen. The porous structure of the films is evident from the SEM images of the top view (Figure 5A) as well as the cross-sectional view (Figure 5B) of the film. Due to edge effects i.e., the concentration of electric field on the substrate edges, the film thickness was highest at the edges as evident in Figure 4A (Pascall et al., 2013). It was also observed that the film roughness increased with time, which could be attributed to the decreasing stability of the suspension as the pHe of the suspension shifts towards the isoelectric point of alumina with time (Tiwari et al., 2020). The decrease in suspension stability leads to the agglomeration of particles in the suspension, which these agglomerates then deposit onto the substrate and increase film roughness Joung and Buie, 2011.

**FIGURE 6:**
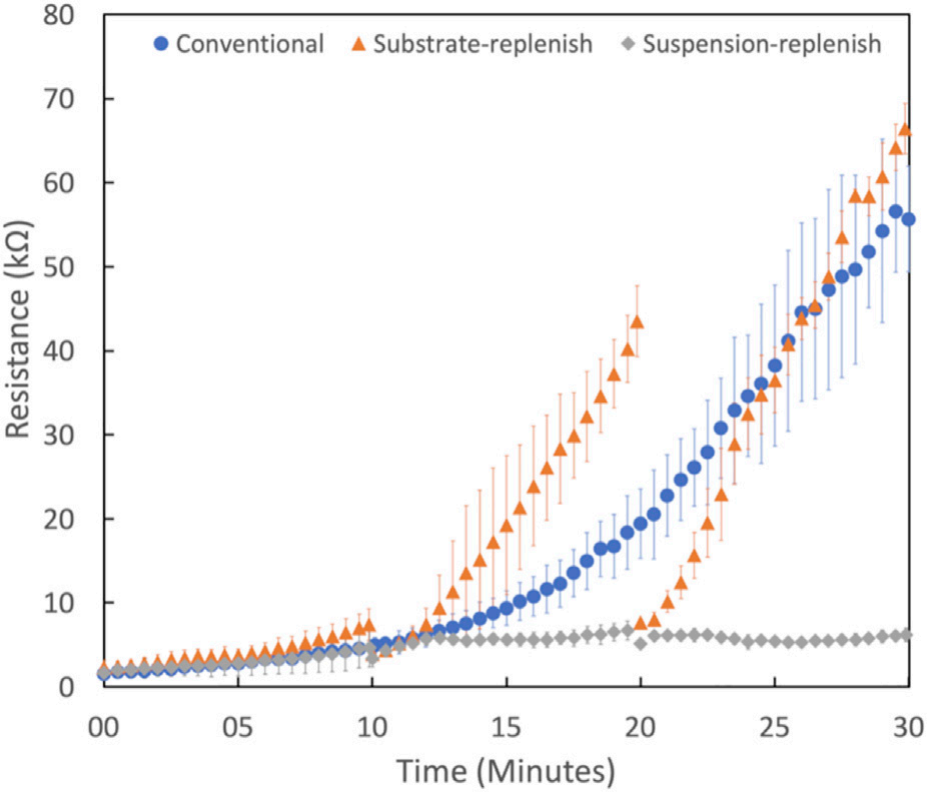
Deposit resistance as a function of time for conventional electrophoretic deposition (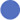) substrate-replenish electrophoretic deposition (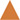) suspension-replenish electrophoretic deposition (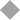) of alumina nanoparticles performed at 100 V/cm in constant-voltage mode. For each type of deposition, three replicate runs were performed to calculate the average and SD in deposit resistance plotted here.

**FIGURE 7:**
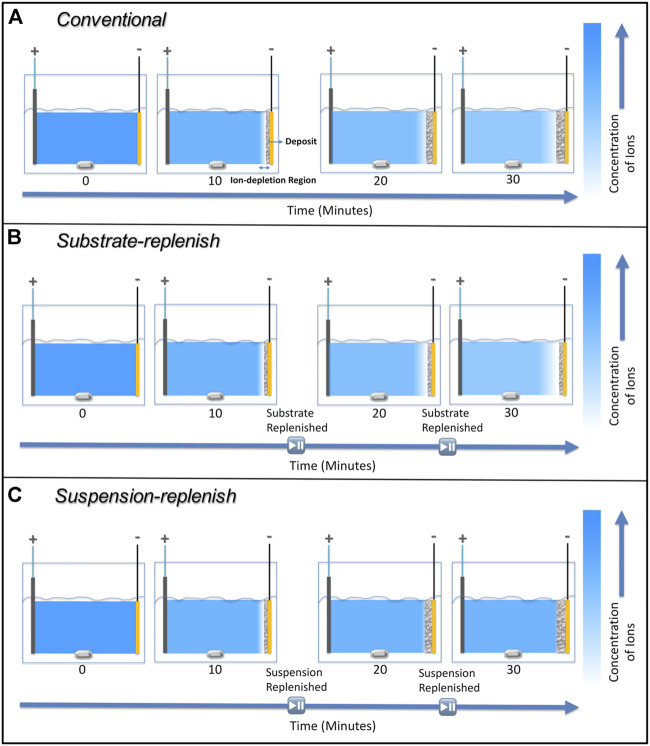
Schematic showing the ion-depletion region and deposit growth on the substrate during **(A)** conventional electrophoretic deposition, **(B)** substrate-replenish electrophoretic deposition, and **(C)** suspension-replenish electrophoretic deposition.

To measure how the deposit resistance changes during conventional electrophoretic deposition, we performed 30-min depositions of the positively charged alumina nanoparticles with an applied electric field of 100 V/cm. Figure 6 shows that the deposit resistance increases exponentially during conventional EPD. This increase can be attributed to the growing thickness of the film of deposited alumina nanoparticles since it has a higher resistivity than the suspension it is replacing (Sarkar and Nicholson, 1996). Secondly, it could be caused due to the formation and growth of a high resistivity ion-depletion region on the substrate. This region is formed because of the depletion of H_3_O^+^ or H_2_EtO^+^ ions as they undergo electrochemical reactions to form H_2_ gas on the substrate (De and Nicholson, 2004; van Tassel and Randall, 2007a). This region is not present on the counter electrode because the primary reactions there result in the generation of ions (H_2_EtO^+^ or H_3_O^+^) while the depletion of Cl^-^ ions as they undergo electrochemical conversion into Cl_2_ is negligible (van Tassel and Randall, 2007b). Therefore, both deposit and ion-depletion regions grow unhindered for the entire duration of the deposition in conventional EPD as shown with the help of a schematic in Figure 7A.

### 3.2 Deposit resistance during substrate-replenish electrophoretic deposition

To isolate the effect of the ion-depletion region growth on the deposit resistance, we performed substrate-replenish EPD (Figure 2A). In this modified EPD approach, we ran 30-min depositions starting with a fresh suspension of alumina nanoparticles at 100 V/cm while pausing the deposition at 10-min and 20-min time points to remove the substrate and introduce a fresh substrate followed by resumption of the deposition. Here, the ion-depletion region is growing for the entire 30-min duration of the experiment, while the deposit growth is restarted every 10 min *via* substrate-replenishment, shown schematically in Figure 7B. Figure 6 shows that the deposit resistance in substrate-replenish EPD increases exponentially from 0 to 10 min and then drops sharply to nearly the original value at 10 min, followed by an exponential rise from 10 to 20 min and drop to nearly the original value at 20 min, and finally an exponential rise from 20 to 30 min. The increase in deposit resistance during substrate-replenish EPD is quite similar to that in conventional EPD (Figure 6) suggesting that the deposit resistance is mainly a function of the ion-depletion region growth.

Note, that there are two minor differences between the deposit resistance increase in conventional and substrate-replenish EPD. First, the rate of the exponential rise in deposit resistance from 10 to 30 min in substrate-replenish EPD is greater than that in conventional EPD, which we attributed to the faster electrochemical depletion of ions in the substrate replenish EPD because of the greater conductive surface area provided by periodic replacement of substrate covered by deposit film with fresh substrates having no deposit on them to begin with. Secondly, the return of deposit resistance deposit to the baseline resistance at 10- and 20-min time-points is attributed to the temporary removal of the ion-depletion region as the suspension is homogenized upon collection in the centrifuge tube.

### 3.3 Deposit resistance during suspension-replenish electrophoretic deposition

Finally**,** to isolate the effect of the growing deposit on the deposit resistance, we performed suspension-replenish EPD (Figure 2B). In this modified EPD approach, we ran 30-min depositions starting with a fresh suspension of alumina nanoparticles at 100 V/cm while pausing the deposition at 10-min and 20-min time points to remove the old suspension and introduce a fresh suspension followed by resumption of the deposition. In this approach, the deposit is growing for the entire 30 min while the ion-depletion region growth is restarted every 10 min with suspension-replenishment as shown with the help of a schematic in Figure 7C. Figure 6 shows that in suspension-replenish EPD, deposit resistance remains nearly constant with time relative to the conventional and substrate-replenish EPD. These results further confirm that the deposit resistance is primarily a function of the growth of the ion-depletion region and not due to the deposit growth. This also proves our hypothesis that the deposit resistance increase can be mitigated by minimizing the growth of the ion-depletion region *via* the replenishment of ions.

In summary, deposit resistance increases by a factor of only 3.7x during suspension-replenish EPD compared to 36x and 29x in conventional and substrate-replenish EPD, respectively. Therefore, it can be concluded that the growth of the ion-depletion region and not the deposit growth is the main driver for the increase in deposit resistance during EPD in agreement with recent studies (Argirusis et al., 2006; Stappers et al., 2008; Schneider et al., 2010). This also confirms our hypothesis that the deposit resistance and hence electric field can be maintained nearly constant by hindering the growth of the ion-depletion region *via* ion replenishment using a novel suspension replenish EPD approach which can help improve EPD yield.

## 4 Conclusion

In this work, we isolated the individual effects of the growth of the ion-depletion region and deposit growth on the local deposit resistance increase during electrophoretic deposition. We found that the main cause for the increase in deposit resistance is the ion-depletion region which forms and grows due to the electrochemical depletion of ions on the substrate. On the other hand, the effect of deposit growth on the increase in deposit resistance was found to be minimal. We also showed that deposit resistance, which is known to be the cause behind the decrease in electric field in the suspension during constant-voltage EPD can be maintained nearly constant *via* ion-replenishment using a new suspension-replenish EPD. While in Stappers et al.’s study, deposit resistance increased with time, which is most likely due to the fact they were recirculating instead of replenishing the suspension (Stappers et al., 2008). Thus, by using a suspension replenish approach the electric field in the suspension can be maintained constant improving EPD yield. Thereby, enabling a route to manufacture nanomaterials-based products and devices in an efficient and scalable manner.

## Data Availability

The original contributions presented in the study are included in the article/supplementary material, further inquiries can be directed to the corresponding author.
